# The Impact of Endoplasmic Reticulum-Associated Protein Modifications, Folding and Degradation on Lung Structure and Function

**DOI:** 10.3389/fphys.2021.665622

**Published:** 2021-05-25

**Authors:** Emily M. Nakada, Rui Sun, Utako Fujii, James G. Martin

**Affiliations:** ^1^Meakins-Christie Laboratories, Research Institute of the McGill University Health Centre (RI-MUHC), McGill University, Montreal, QC, Canada; ^2^McGill University, Montreal, QC, Canada

**Keywords:** unfolded protein response, endoplasmic reticulum, integrated stress response, post-translational modifications, disulfide bonds, lung disease, lung function

## Abstract

The accumulation of unfolded/misfolded proteins in the endoplasmic reticulum (ER) causes ER stress and induces the unfolded protein response (UPR) and other mechanisms to restore ER homeostasis, including translational shutdown, increased targeting of mRNAs for degradation by the IRE1-dependent decay pathway, selective translation of proteins that contribute to the protein folding capacity of the ER, and activation of the ER-associated degradation machinery. When ER stress is excessive or prolonged and these mechanisms fail to restore proteostasis, the UPR triggers the cell to undergo apoptosis. This review also examines the overlooked role of post-translational modifications and their roles in protein processing and effects on ER stress and the UPR. Finally, these effects are examined in the context of lung structure, function, and disease.

## Endoplasmic Reticulum Stress and the Unfolded Protein Response

Cells are normally in a state of proteostasis, whereby networks of signaling pathways work in concert to maintain the proper synthesis, folding, trafficking, and degradation of proteins. It is thought that a third of all proteins traffic through the endoplasmic reticulum (ER) for post-translational modifications (PTMs), folding, and trafficking (Huh et al., 2003). Under pathological or even physiological conditions, as well as in response to chronic stimuli, there is likely to be an accumulation of misfolded or unfolded proteins in the ER. This accumulation is referred to as ER stress and leads to the activation of the unfolded protein response (UPR) that inhibits *de novo* protein synthesis, while permitting the expression of protein-folding machinery and increasing degradation of unfolded proteins. If effective, the UPR attenuates ER stress and avoids cellular apoptosis (Hetz et al., 2015). Protein degradation or autophagy is an essential counterpart of protein synthesis and inhibition or a defect in autophagy leads to cell swelling. Autophagy is regulated by complex mechanisms which include pathways affecting cell metabolism, division, and autophagy, including the mevalonate pathway (Miettinen and Bjorklund, 2015). Further consideration of these pathways, however, is beyond the scope of this review.

## The UPR Sensors

The UPR is a highly conserved response consisting of the three canonical receptors, protein kinase R-like ER kinase (PERK), inositol-requiring enzyme (IRE)1α, and activating transcription factor (ATF)6α, as well as the mediators that comprise each of their downstream signaling pathways (Hetz et al., 2015). Glucose-regulated protein 78 kDa (GRP78; binding immunoglobulin protein) binds all three receptors on the luminal surface of the ER membrane, where it acts as the master regulator of the UPR (Bertolotti et al., 2000; Shen et al., 2002). It simultaneously functions as a chaperone, directly aiding in the proper folding of unfolded proteins. Interestingly, in its role as a chaperone, GRP78 acts as the central regulator of the UPR. In response to ER stress, less GRP78 is bound to PERK, IRE1α, and ATF6α as it preferentially aids in the proper folding of proteins (Sundaram et al., 2018). GRP78 binds proteins with high promiscuity, recognizing and preferentially binding sequences containing hydrophobic amino acids that ordinarily would not be exposed in their properly folded state (Flynn et al., 1991). Thus, under conditions of high ER stress, GRP78 preferentially binds to unfolded proteins accumulating in the lumen of the ER, leaving PERK, IRE1α, and ATF6α unbound and in an active state. Consequently, all three receptors trigger mechanisms responsible for decreasing the unfolded protein load in an effort to return the cell to protein homeostasis. In contrast, under unstressed conditions, there are few unfolded proteins in the ER lumen, which consequently liberates GRP78 to bind the UPR receptors, which maintains them in their inactive state.

### Inositol-requiring Enzyme 1α

**Figure 1:**
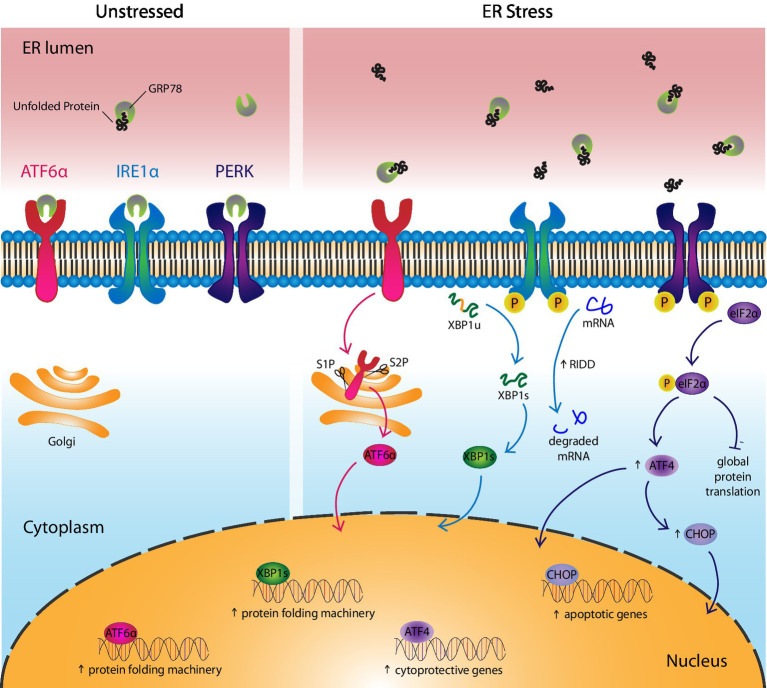
Cells respond to ER stress *via* UPR activation. The UPR consists of three canonical receptors, activating transcription factor 6α (ATF6α), inositol-requiring enzyme 1α (IRE1α), and protein kinase R-like ER kinase (PERK). (Left) Under unstressed conditions, GRP78, the UPR ligand and chaperone, binds the UPR receptors to maintain them in their inactive state. (Right) In response to ER stress, GRP78 leaves the UPR receptors and assists in the folding of unfolded proteins, thereby activating the receptors. More specifically, ATF6α translocates to the Golgi where it is cleaved by enzymes, site-1 protease and site-2 protease, before entering the nucleus as a transcription factor that upregulates genes involved in protein folding. IRE1α undergoes autophosphorylation and activates its endoribonuclease, which splices unspliced XBP1(u) mRNA into spliced XBP1(s). XBP1s protein acts as a transcription factor that upregulates genes involved in protein folding. IRE1α also reduces ER stress by splicing mRNAs into non-functional transcripts before they can be translated, a process called regulated IRE1-dependent decay. PERK autophosphorylates then phosphorylates eIF2α, which inhibits protein translation, with the exception of ATF4-regulated genes like CHOP. ATF4 upregulates cytoprotective genes and in the case of chronic ER stress, it induces apoptosis *via* CHOP.

Inositol-requiring enzyme1α, first discovered in 1993, is a type I transmembrane receptor consisting of both a protein kinase domain and an endoribonuclease domain that targets and splices out a sequence of 26 base pairs from the introns of mammalian X-box binding protein-1 (*Xbp1*) (Cox et al., 1993). This takes place following IRE1α disengagement from GRP78, its dimerization/oligomerization, autophosphorylation, conformational change, and activation of its ribonuclease (Figure 1). Spliced XBP1 (XBP1s), translated from *Xbp1s* mRNA, acts as a transcription factor that regulates the expression of ER stress-reducing chaperones and enzymes. Genes regulated by XBP1s are also involved in protein trafficking in and out of the ER and post-translational modifications (PTMs; Tirasophon et al., 1998; Tirosh et al., 2006; Oikawa et al., 2009). In contrast, unspliced XBP1 protein acts as a dominant-negative regulator of the UPR (Lee et al., 2003).

There is also an XBP1-independent pathway downstream of the IRE1α sensor that regulates the UPR. In chronic ER stress, expression of XBP1 is reduced. The endoribonuclease activity of IRE1α switches from targeting *Xbp1* for splicing to selectively degrading mRNAs that produce proteins that are notoriously difficult to fold accurately under ER stress conditions (Hollien and Weissman, 2006; Hollien et al., 2009; Hetz et al., 2011). This process is called the regulated IRE1-dependent decay (RIDD) pathway. This pathway alleviates ER stress by degrading mRNAs in the cytoplasm, before they can be translated into nascent proteins that aggravate ER stress. Finally, IRE1α plays an important role in the degradation of misfolded proteins *via* ER-associated degradation (ERAD), which is discussed in detail in the ERAD section of this review.

### Activating Transcription Factor 6α

Unlike IRE1α, ATF6α is a type II transmembrane receptor first characterized as a UPR sensor in 1999 (Haze et al., 1999). ATF6α consists of a transmembrane domain that anchors it to the ER, a luminal domain that binds GRP78, and a basic leucine-zipper domain facing the cytoplasm (Hetz, 2012). The receptor is constitutively expressed as a 90 kDa protein that translocates to the Golgi after the release of GRP78, where it is activated following its cleavage into a 50 kDa product by site-1 protease (S1P) and site-2 protease (S2P; Figure 1; Ye et al., 2000; Shen et al., 2002). Activated ATF6α binds to ER stress-associated promoter sites in the nucleus to positively regulate ER stress-responsive genes like GRP78, GRP94, and calreticulin (CRT). It recognizes the ER stress response element with the sequence, CCAAT-N_9_-CCACG, in the promoter regions of these genes (Yoshida et al., 1998). Notably, ATF6β, which is also upregulated in response to ER stress, acts as a dominant negative regulator and may provide a mechanism through which the intensity and duration of ATF6α activation is regulated (Thuerauf et al., 2004). Although ATF6α is not essential for embryonic or post-natal development and despite considerable overlap in function with the other two UPR receptors, the importance of ATF6α appears central in orchestrating an effective chronic adaptive response to ER stress (Wu et al., 2007).

### Protein Kinase R-like ER Kinase

Protein kinase R-like ER kinase is a type I transmembrane receptor that initiates a global reduction in protein synthesis, while selectively translating transcripts that extend the protein-folding capacity of the ER. PERK was first described in 1998 as a UPR sensor consisting of a luminal domain that binds GRP78, a transmembrane domain that traverses the ER membrane, and a cytoplasmic tail with protein kinase activity (Shi et al., 1998; Harding et al., 1999). Under ER stress conditions, PERK is released by GRP78, causing it to dimerize, autophosphorylate, and undergo a conformational change before phosphorylating eukaryotic initiation factor-2α (eIF2α; Figure 1). Phosphorylated (P)-eIF2α reduces protein translation by the competitive inhibition of eIF2β, a key component of an essential complex required in the initiation step of protein translation that allows transfer RNA binding to the AUG start codon (Gebauer and Hentze, 2004).

While P-eIF2α decreases global protein synthesis, it promotes the translation of select transcripts through alternative mechanisms like internal ribosomal entry sites or by bypassing inhibitory open reading frames (ORFs) upstream of target genes, as is the case with accessing the start codon of the *Atf4* ORF (Harding et al., 2003; Ameri and Harris, 2008; Singleton and Harris, 2012). ATF4 regulates transcription of genes involved in cell metabolism, oxidative stress, and amino acid transport by binding C/ebp-Atf response element sequences of targeted genes (Kilberg et al., 2009). Many ATF4-regulated genes empower cells to respond to ER stress by increasing the protein folding capacity of the cell, including activating ATF6α by assisting in its synthesis and trafficking from the ER to the Golgi (Teske et al., 2011). However, under chronic ER stress conditions, the cell can undergo apoptosis through ATF4 upregulation of C/EBP Homologous Protein (CHOP) as part of the PERK-eIF2α-ATF4-CHOP axis. The details of this process are discussed in detail in the next section of the review.

## Apoptosis

Although the cell responds to ER stress by increasing the protein-folding capacity of the cell, degrading misfolded/unfolded proteins, and decreasing *de novo* protein synthesis, the UPR can fall short of its ability to return the cell to proteostasis. Unalleviated ER stress-induced chronic UPR activation positively regulates CHOP expression to signal cellular apoptosis (Hu et al., 2018). CHOP, also known as growth arrest and DNA damage-inducible gene 153, is a transcription factor that is upregulated by the PERK-eIF2α-ATF4 axis, following ATF4-binding of the C/ebp-Atf response element sequence in its promoter. The IRE1α and ATF6α pathways of the UPR can also contribute to CHOP expression, but play secondary roles to that of PERK (Li et al., 2014).

C/EBP Homologous Protein consists of two functional domains, an N-terminal transcriptional activation domain and a C-terminal basic leucine zipper domain (Ubeda et al., 1996). CHOP functions by upregulating expression of pro-apoptotic and downregulating expression of anti-apoptotic members of the B cell lymphoma (BCL)2-family of proteins (Li et al., 2014). CHOP has been shown to downregulate expression of anti-apoptotic BCL2, BCL-XL, and myeloid cell leukemia sequence 1, while upregulating pro-apoptotic Bcl-2-like protein 11, which increases Bcl-2 homologous antagonist killer and Bcl-2-associated X (BAX) expression. BAX leads to the permeabilization of the mitochondrial membrane through the formation of permeability transition pores, allowing cytochrome c, second mitochondria-derived activator of caspases, apoptosis-inducing factor, and other apoptotic substrates to enter the cytoplasm where apoptosomes are formed. Activation of caspase-9, a component of the apoptosome, leads to the activation of execution caspases like caspases-3, -6, and -7, which cleave macromolecules and activate cytoplasmic endoribonucleases that proceed to cleave DNA, causing cell death (Siddiqui et al., 2015; Hu et al., 2018).

C/EBP Homologous Protein may also induce cell death through alternative mechanisms. Although controversial, studies show CHOP-induced death receptor 5 accumulation in the ER, in response to ER stress. This leads to caspase-8 activation, followed by the formation of the death-inducing signaling complex, which converges on activating the execution caspases to mediate apoptosis (Moon et al., 2013; Lu et al., 2014; Glab et al., 2017). CHOP-induced ER oxidoreductase (ERO)1α is another mechanism converging on the executing caspases that signal cell death. In a model of acute liver failure, ERO1α activity was shown to produce high levels of H_2_O_2_ in the process of oxidizing protein disulfide isomerases (PDIs) (Rao et al., 2015). PDIs are essential to the formation of disulfide bonds for proper protein folding and are therefore in high demand during ER stress. Thus, a potential consequence of accurately folding more proteins may be in elevating the production of H_2_O_2_, which could leak into the cytoplasm where it signals cell death through caspase-3.

## ER-Associated Degradation

Because one-third of all proteins that traffic to the ER never exit the organelle as a functional protein, a mechanism of rapidly degrading terminally misfolded proteins in the ER is necessary. It is through ERAD that the proteins are retro-translocated to the cytoplasm where they are degraded. ERAD has long been considered a part of the UPR, as the expression of many of its mediators are regulated by UPR transcription factors; however, recent studies suggest that it is a separate protein quality control pathway that communicates closely with the UPR to maintain cellular proteostasis (Hwang and Qi, 2018). More specifically, IRE1α was recently determined to be an endogenous substrate of ERAD, making the ERAD machinery as much a regulator of, as it is regulated by, the UPR (Sun et al., 2015).

**Figure 2:**
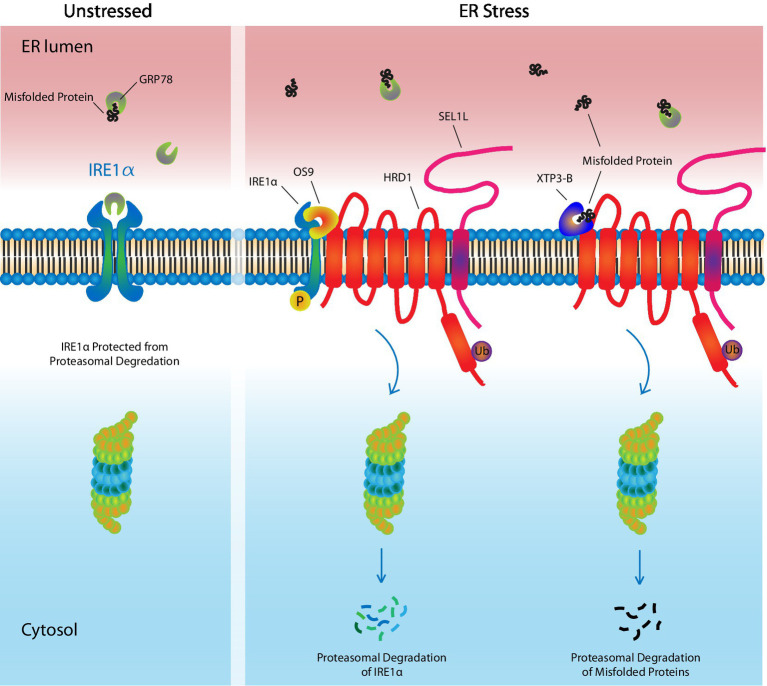
ER-Associated Degradation. Under unstressed conditions, the UPR receptor, IRE1α, is bound by the UPR ligand and chaperone, GRP78, which maintains it in an inactivate state, while also protecting it from targeted degradation by ERAD machinery. In response to ER stress, GRP78 leaves IRE1α and preferentially binds misfolded/unfolded proteins, which have accumulated in the ER lumen. This leaves IRE1α unprotected. OS9 and XTP3-B assist in targeting IRE1α, as well as misfolded proteins, to the HRD1/SEL1L complex where they undergo ubiquitinylation followed by proteasomal degradation, thereby reducing ER stress. Ub, ubiquitin.

The ERAD machinery functions by first selecting appropriate substrates for degradation, followed by their retro-translocation to the cytosol, ubiquitinylation, and finally their proteasomal degradation (Figure 2). ERAD substrates are selected by OS9 or XTP3-B, lectin proteins that engage misfolded proteins and IRE1α, with the SEL1L-HRD1 ERAD complex (Fujimori et al., 2013; Sun et al., 2015). GRP78 competes with ERAD for these substrates, such that under basal conditions, GRP78-bound IRE1α is protected and stably expressed, whereas under ER stress, unbound IRE1α is more easily targeted for degradation. SEL1L is a cofactor for HRD1, a multi-spanning ubiquitin E3 ligase that forms a channel through the ER membrane through which ERAD substrates are transported to the cytoplasm. Substrate interaction with the SEL1L-HRD1 complex leads to its polyubiquitinylation, followed by proteasomal degradation (Sun et al., 2015; Wu and Rapoport, 2018; Li et al., 2020). In some instances, misfolded proteins that are too large, such as protein aggregates, are eliminated by ER-to-lysosome-associated degradation (Li et al., 2020). As the name suggests, these aggregates are engulfed by vesicles that are delivered to lysosomes where they are degraded.

## Post-Translational Modifications

Approximately 5 amino acids are translated per second by each ribosome, with almost 2 min devoted to fully translating the average 438 residue protein in eukaryotic cells. In contrast, the average half time to properly fold proteins is between 30 and 60 min and takes an average of 1–2 h for proteins to be secreted (Braakman and Hebert, 2013; Sharma et al., 2019). So, while nascent proteins quickly enter the ER, the time-consuming course of protein-folding allows unfolded proteins to rapidly accumulate, in the absence of adequate protein-folding machinery. Protein folding can be a slow process because of the PTMs that proteins in the ER undergo, including signal peptide (SP) removal, N-linked glycosylation, disulfide bond (S–S) formation, palmitoylation, and proline hydroxylation (Ellgaard et al., 2016). In addition, many proteins, including most membrane glycoproteins and extracellular matrix proteins, undergo full or partial oligomerization in the ER before secretion (Hurtley and Helenius, 1989). This review will briefly summarize the three most common and well understood PTMs that occur in the ER, which are SP removal, N-linked glycosylation and S–S formation.

### Signal Peptide Cleavage

The importance of the ER to the proper functioning of proteins cannot be overstated. The role of the ER begins with the SP, a short peptide sequence, generally within the first 25 amino acids translated by a ribosome that traffics novel proteins to specific organelles (Petersen et al., 2011). Although short in sequence, the SP consists of a hydrophobic N-terminal basic domain, a hydrophobic domain, and a polar domain containing the cleavage site (Hegde and Bernstein, 2006). The N-terminal and hydrophobic domains help position the peptide in a looped configuration during translocation to the ER. The cleavage-domain is oriented to face the lumen for immediate recognition and cleavage by the signal peptidase complex on the ER where translation continues (O’brien et al., 2014). The SP sequence can affect the efficiency of peptide cleavage, its maturation, and targeting, the last of which explains why some mature proteins can be directed to two distinct areas of the cell, such as CRT, which is co-localized to both the ER and the cytoplasm (Shaffer et al., 2005).

### N-linked Glycosylation

Asparagine (N)-linked glycosylation is a highly conserved PTM with most secreted proteins from eukaryotic cells undergoing the alteration. In addition to its importance in protein folding, N-linked glycosylation is fundamental for molecular recognition, cell–cell communication, and protein stability (Braakman and Hebert, 2013; Mohanty et al., 2020). The enzymatic reaction involves the transfer of an oligosaccharide group from a donor substrate (lipid-linked oligosaccharide) to the acceptor substrate (asparagine residue) on newly synthesized proteins by the membrane-associated complex, oligosaccharyltransferase. Once transferred, N-linked oligosaccharides must be trimmed by glucosidases 1 and 2 to acquire a monoglucosylated glycan that can be recognized by the ER lectin molecules, calnexin (CNX) and CRT (Cherepanova et al., 2016). The lectin chaperones increase the efficiency of glycoprotein folding, prevent protein aggregation and premature exiting of the ER, and decrease misfolding by slowing down the kinetics of protein folding (Helenius, 1994; Price et al., 2012). The lectin chaperones recruit the oxidoreductase, PDI family A, member 3 (PDIA3; ERP57), and the peptidyl-prolyl isomerase, cyclophylin B, to assist in protein folding. Oligosaccharides on glycoproteins released by CNX and CRT may then be trimmed of a mannose residue by ER mannosidase I, before the glycoprotein is secreted or takes up permanent residence in the ER (Cherepanova et al., 2016). An error in N-linked glycosylation or excessive, sequential mannose trimming by ER degradation-enhancing α-mannosidases 1, 2 and 3, can lead to targeting of the misfolded glycoprotein for ERAD.

### Disulfide Bond Formation

**Figure 3:**
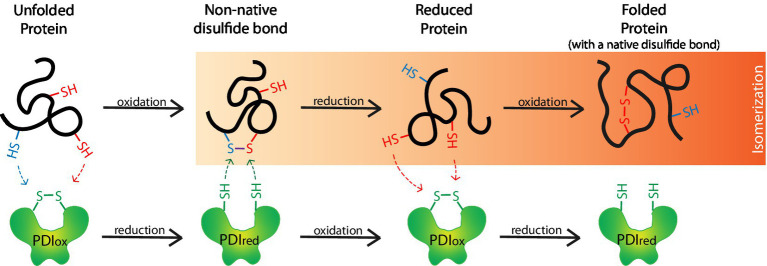
Protein disulfide isomerases (PDIs) form disulfide bridges that assist in the proper folding of proteins. PDIs (PDIox) oxidize thiol/sulfhydryl (–SH) side chains on unfolded proteins to form disulfide bonds (S–S) and are thereby reduced (PDIred). S–Ss often form between incorrect thiols (i.e., blue-SH with a red-SH) to form non-native S–Ss. When this occurs, the S–S undergoes isomerization whereby non-native S–Ss are reduced back to-SHs by a PDIred. A PDIox then oxidizes the correct-SHs (i.e., 2 red-SHs) on the reduced protein to form the correct native S–S and produce a properly folded protein.

Oxidoreductases are enzymes that catalyze the transfer of electrons from one molecule, the donor/reductant, to another, the acceptor/oxidant. PDIs are thiol oxidoreductases that are essential in properly folding S–S-containing proteins. 29.5% of eukaryotic proteins are predicted to contain a S–S. While peptides of moderate length between 100 and 400 amino acids average less than 1 S–S, peptides less than 100 amino acids average a single bond, and large peptides with >400 amino acids average two bonds (Bosnjak et al., 2014). PDIs are involved in the formation, breakdown, and rearrangement of these bonds, meaning they oxidize, reduce, and isomerize S–Ss, respectively. During the formation of the disulfide bridges, PDIs oxidize thiol/sulfhydryl side chains (–SHs) on cysteine residues within and between peptide(s) to form intramolecular and intermolecular S–Ss, respectively (Figure 3; Ellgaard and Ruddock, 2005; Braakman and Hebert, 2013). These bonds often undergo isomerization before the protein achieves its final conformation. This involves an oxidized PDI that forms the initial bond, followed by the action of a reduced PDI that reduces the bond between the incorrect cysteine residues, before the now re-oxidized PDI can catalyze the new bond formation between the correct residues. These bonds help stabilize proteins in their correct tertiary and/or quaternary structures. To efficiently oxidize-SHs, PDIs require a highly oxidative environment like the ER lumen. In this environment, the oxidoreductase ERO1α can continuously re-oxidize PDIs (Appenzeller-Herzog, 2011). Recently, alternatives to ERO1α have been identified as PDI oxidants, including peroxiredoxin 4 and vitamin K epoxide reductase, but will not be discussed further (Wajih et al., 2007; Tavender et al., 2010).

More than 20 mammalian PDIs have been discovered that vary in their domains and activity, but all have at least one thioredoxin (Trx)-like domain. The number, location, redox potential, orientation, and electrostatic potential of their domains determine PDI function, including their ability to form, reduce and isomerize S–Ss, bind ERO1α and other substrates, retain proteins in the ER, traffic terminally misfolded proteins to the cytosol for proteasomal degradation, and whether they have chaperone activity (Okumura et al., 2015; Soares Moretti and Martins Laurindo, 2017). PDIA1, also simply referred to as PDI, was the first to be discovered and although ubiquitously expressed, is more highly expressed in secretory cells (Edman et al., 1985). It consists of four Trx-like domains (a, b, b' and a', starting from the N-terminus) in a “U” shape, with only the terminal ends having the catalytically active sequence Cys-X-X-Cys, and the b' domain binding substrate. PDIA1 in the oxidized state has a more open conformation compared to its reduced state, which could explain its ability to efficiently form disulfide bridges within and between a wide-range of substrates, bringing cysteine residues in close proximity to one another (Okumura et al., 2015). In contrast, a PDI like Erp27 is comprised of two non-catalytically active Trx-like domains, b and b', and is thought to bind and bring misfolded proteins to catalytically active PDIs like PDIA3 for S–S formation (Kober et al., 2013). Ultimately, PDIs are positively regulated by the UPR and contribute to the protein-folding machinery of the cell to attenuate ER stress.

## Protein Processing in Lung Structure and Function

ER stress can occur under physiological conditions, including the G2/M phase of the cell cycle, in cells undergoing differentiation, and in secretory cells that continuously work on the maturation of proteins destined for secretion (Matsuzaki et al., 2015; Lee et al., 2019). However, acute and chronic ER stress, induced by endogenous and exogenous sources can challenge cells to return to proteostasis and may ultimately be detrimental to the proper functioning of cells, tissues, and organs. Tunicamycin (Tm), a chemical that induces ER stress by inhibiting N-linked glycosylation of proteins, has been administered to animals to study the effects of ER stress on the lungs. Tm was shown to worsen airway inflammation in an animal model of sepsis, enhance neutrophilic inflammation and airway hyperresponsiveness (AHR) in an ovalbumin-lipopolysaccharide model of asthma, and enhanced bleomycin-induced fibrosis (Lawson et al., 2011; Guo et al., 2017; Chen et al., 2020). Thus, augmenting ER stress in airway disease models in which ER stress is intrinsic to the disease, can worsen pathology.

Understanding the role of ER stress and the UPR can be difficult and is further complicated by the lack of methodology to quantify ER stress, considering the difficulty in producing a reliable reagent that can recognize all unfolded and misfolded proteins. Currently, the most reliable method measures ER dilation, typically by visualizing the expanded lumen of the ER by electron microscopy (Oslowski and Urano, 2011). Alternatively, mediators of the UPR, which are upregulated and/or activated in response to ER stress, are measured. However, because the UPR is a response to ER stress and not a direct measurement, it is important to correctly interpret the data. For example, an increase in the expression of GRP78 in the lungs of bleomycin-exposed mice would indicate an increase in ER stress. Deterioration of the disease in mice pre-treated with a siRNA targeting GRP78 could be due to either an increase or decrease in ER stress, following a decrease in chaperone activity provided by GRP78 or an increase in activation of the UPR with inadequate GRP78 to bind/inactivate the receptors, respectively. Thus, it is imperative that the role of ER stress and the UPR be interpreted alongside additional UPR mediators and readouts to discern whether a specific mediator of or the UPR in general plays a beneficial or harmful role in the pathogenesis of a disease.

### Extracellular Matrix

Inhibition of the IRE1α pathway has been shown to improve TGFβ1-induced collagen and fibronectin production by fibroblasts from patients with idiopathic pulmonary fibrosis (IPF), cytokine-induced mucus production in human airway epithelial cells (AECs), and mucus production in the distal murine airway epithelia in murine models of fibrosis (Ghavami et al., 2018; Chen et al., 2019). GRP78 deficient mice showed greater airway remodeling, fibrosis, inflammation and mortality in one study, while CHOP deficient mice were protected from lung fibrosis in several murine models of fibrosis, including a bleomycin-induced model (Burman et al., 2018a; Borok et al., 2020). Thus, consistent with results from airway disease studies, GRP78 is likely to be protective, while CHOP expression may be damaging in IPF.

Idiopathic pulmonary fibrosis is a serious and often fatal interstitial lung disease characterized by fibrotic airway remodeling, progressive dyspnea, and respiratory failure (Burman et al., 2018b). Aberrant fibroblast, type II alveolar epithelial cell, and inflammatory cell activity are implicated in IPF progression. ER stress was first implicated in IPF with the discovery of mutations in surfactant protein C, a major protein secreted by type II alveolar epithelial cells, which can result in misfolding (Nogee et al., 2001). Since these cells are secretory in function, mutations in surfactant protein C can further elevate ER stress in these cells. The UPR markers GRP78, ERAD-enhancing α-mannosidase-like proteins, XBP1, CHOP, ATF4 and ATF6 have been detected at elevated levels in the lungs of IPF patients, especially in alveolar type II epithelial cells (Korfei et al., 2008; Lawson et al., 2008). These were accompanied by the increase in activation of pro-apoptotic pathways, specifically the cleavage of Bax and caspase-9. In addition, ER stress also promotes the epithelial to mesenchymal transition of alveolar type II epithelial cells, potentially contributing to the pool of pulmonary fibroblasts (PFs), culminating in the excessive deposition of extracurricular matrix (ECM; Tanjore et al., 2015; Kropski and Blackwell, 2018).

PFs are the primary cells responsible for the maintenance of healthy ECM in the parenchyma and disorders in their function can result in their differentiation into myofibroblasts, accompanied by the excessive production of ECM proteins and the stiffening and distortion of tissue as observed in interstitial lung diseases (Burman et al., 2018b). The elevated ER stress in PFs is associated with increased expression of GRP78 and all three of its receptors in PFs derived from IPF patients (Baek et al., 2012). TGFβ, the major growth factor that stimulates PF biosynthesis of ECM and differentiation into myofibroblasts, upregulates GRP78 and activates the IRE1α-XBP1 and ATF6α pathways in human PFs, which is in part due to oxidative stress (Baek et al., 2012; Ghavami et al., 2018). Inhibition of oxidative stress in cultured fibroblasts, using glutathione or N-acetyl cysteine, reduced TGFβ-induced GRP78, α-smooth muscle actin and type I collagen expression (Baek et al., 2012) Inhibition of ER stress with 4-phenylbutyric acid or GRP78 knock-down also reduced TGFβ-induced α-smooth muscle actin (αSMA) and type I collagen expression, while an IRE1 inhibitor alleviated TGFβ-induced myofibroblast differentiation and reduced their biosynthesis of collagen and fibronectin (Baek et al., 2012; Ghavami et al., 2018). In general, IRE1α activation drives myofibroblast differentiation by cleaving miR-150, a miRNA that suppresses αSMA expression (Heindryckx et al., 2016). In a bleomycin-induced murine model of fibrosis, an elevation in ER stress resulted in the activation of all three UPR-associated receptors in the whole lung and PFs, which was associated with PF proliferation and excessive collagen deposition (Baek et al., 2012; Hsu et al., 2017; Thamsen et al., 2019). ER stress inhibitors, tauroursodeoxycholic acid and 4-phenylbutyric acid inhibited PF proliferation through the reduced activation of the PI3K/AKT/mTOR pathway, subsequently ameliorating fibrosis and improving lung function (Hsu et al., 2017). Similarly, IRE1α-specific inhibition resulted in reduced lung collagen, hydroxyproline content and reversed bleomycin-induced fibrosis in mice (Thamsen et al., 2019).

### Barrier Function

The primary role of AECs is to provide a physical barrier between the external environment and the inner milieu. This is accomplished through the mucociliary clearance (MCC) of inhaled microbes and small particles, the production and release of antimicrobial agents, and intercellular adherens and tight junctions (Ganesan et al., 2013). Adherens and tight junctions are located on the apicolateral membrane of epithelial cells and maintain contact with neighboring cells (Hartsock and Nelson, 2008). Tight junctions regulate the transport of ions and solutes in the intercellular space and consist of the transmembrane proteins, occludin and claudin, as well as scaffolding proteins zonula occludens-1, -2, and -3. The adherens junctions form, mature, and maintain cell-to-cell contact with adjacent cells and consist of the transmembrane proteins, E-cadherin and several intracellular proteins including p120-, α- and β-catenins.

In a murine model of allergic airways disease, the ER stress inhibitor, 4-phenylbutyric acid, prevented the allergen-induced loss of epithelial barrier function, represented by losses in zonula occludens-1 and E-cadherin expression, as well as reversing IL-25-induced loss of zonula occludens-1, E-cadherin, and trans-epithelial electrical resistance of 16HBE cells (Yuan et al., 2018). Although very little else is known about the role of ER stress and the UPR in maintaining the airway epithelial barrier, studies in other organ systems indicate that ER stress disrupts tight junctions between human retinal pigment epithelial cells and intestinal epithelial cells (Ma et al., 2016; Huang et al., 2019). Altogether, ER stress appears to decrease the protective physical barrier provided by tight and adherens junctions on epithelial cells and inhibiting allergen-induced loss of these junctions can be reversed using ER stress inhibitors.

### Mucociliary Clearance

Mucociliary clearance is an innate defense mechanism of the lungs that is mediated by AECs and submucosal glands. The role of MCC is to trap, transport, and remove foreign particles, such as pollutants, pathogens, and allergens from the airways. Defects lead to bronchiectasis and other airway pathologies (Chalmers et al., 2018; Nikolic, 2018; Lucas et al., 2020). Cigarette smoke impairs mucociliary transport by inducing airway dehydration and increasing mucus viscosity, both *in vitro* and *in vivo* (Lin et al., 2020). ER stress has not yet been directly implicated. MCC function may be considered in three parts, namely, water and ion transport, mucin production, and ciliary beating. There is a variety of ion channels, including the cystic fibrosis (CF) transmembrane conductance regulator (CFTR), that allow AECs to control the trans-epithelial water flow and as such, controls the viscosity of the mucus and efficacy of the MCC (Hollenhorst et al., 2011). A deficiency or defect in CFTR expression on AECs can lead to severe respiratory impairment due to reduced Cl^-^ secretion, resulting in increased Na^+^ reabsorption and excessive water absorption from fluid on the airway surface (Burch et al., 1995; Mall et al., 1998; Matsui et al., 1998). The increased viscosity and reduced MCC increase CF patient susceptibility to colonization by microbes like *Staphylococcus aureus* and *Pseudomonas aeruginosa*, resulting in chronic inflammation and airway damage (Cantin et al., 2015). Some proteins, like the CFTR, require the PTM, palmitoylation, before they can leave the ER. Preventing palmitoylation of the CFTR is known to inhibit the steady-state expression of both wild type and F508del mutant CFTR and reduce ion channel function on the surface of the cell. When corrected with specific protein acyl transferases regulating palmitoylation, CFTR expression is restored (Mcclure et al., 2014). Accumulating research also indicates that the IRE1α-XBP1 pathway of the UPR is more active in the lungs of CF patients, which promotes expression of pro-inflammatory cytokines like IL-8 (Ribeiro et al., 2005). Cultured human bronchial epithelial cells and alveolar macrophages from CF lungs express more XBP1s compared to controls and the inhibition of IRE1α decreases LPS-induced pro-inflammatory cytokine production in cultured CF cells.

The mucin component of the MCC traps pathogens and small particles. There are at least 21 genes encoding human mucins, 13 of which are expressed in AECs (Ma et al., 2018). Mucin maturation is achieved through an array of PTMs initiated in the ER where nascent mucin monomers undergo N-glycosylation before dimerizing and moving to the Golgi apparatus for O-glycosylation and higher-order multimerization (Ribeiro et al., 2005; Mcclure et al., 2014; Ma et al., 2018). Moreover IRE1β, acting through XBP1, is required for mucin production and its expression in bronchial epithelial cells is upregulated in the airways of asthmatics and CF patients (Martino et al., 2013). Expression of the ER molecular chaperone and member of the PDI family of ER proteins, Anterior Gradient (Agr)2, is increased with MUC5AC expression in the airway epithelium of Th2-high asthmatics and the lungs of mice with allergic airways disease (Schroeder et al., 2012). Immature MUC5AC can be found complexed with Agr2 and its expression reduced by 50% in allergen-challenged Agr2^−/−^ mice. While its overall expression is reduced in Agr2^−/−^ mice, more mucins accumulate at the level of the ER in these mice, where they contribute to ER stress. Tm-induced ER stress induces expression of MUC5AC and MUC5B through XBP1, ATF6α, and CHOP in human nasal epithelial cells (Kim et al., 2019).

Little is known about the contributions of ER stress and the UPR to ciliary beating. Ciliated cells are responsible for the propulsion of the mucus layer that covers the AECs. Proper ciliary function is absolutely required for effective MCC. In the apical region, there is an abundance of mitochondria that ensures ATP availability to sustain ciliary motion. Although its expression is not induced in response to ER stress, the PDI family member AGR3, is an ER resident protein that is uniquely expressed in ciliated cells of the airway epithelium where it regulates the ciliary beat frequency (Bonser et al., 2015). Agr3^−/−^ mice have lower ciliary beat frequencies at baseline and in response to ATP. Finally, it is likely that ER stress would have adverse effects on the integrity of cilia, as their proteins require significant and various PTMs (Wloga et al., 2017).

### Antioxidant Response

The lungs are exposed to thousands of liters of inhaled air each day that contain compounds with the potential to induce oxidative stress, defined as an imbalance of anti-and pro-oxidants. More specifically, oxidative stress can occur following exposure to exogenous oxidizing compounds like ozone, nitrous oxide, and chlorine, or endogenous sources like inflammatory cells that are recruited to the airways following exposure to particulates, allergens or microbes, but may also come from metabolically dysregulated cells or cells with a reduced anti-oxidant capacity (Knaapen et al., 2006; White and Martin, 2010; Kumarathasan et al., 2015). Cells respond to oxidative stress by means of an evolutionarily conserved antioxidant defense mechanism that is predominantly regulated by the transcription factor, nuclear factor erythroid 2-related factor (Nrf)2, and its endogenous inhibitor, Kelch-like ECH associated protein (Keap)1. Under normal conditions, Keap1 binds Nrf2 to sequester it in the cytoplasm and enhance its degradation. During oxidative stress, when electrophiles and reactive oxygen species (ROS) are present, Keap1 is dissociated from Nrf2, allowing Nrf2 to escape degradation and translocate to the nucleus where it positively regulates the expression of anti-oxidant genes like hemeoxygenase-1, the glutathione peroxidases, glutathione S-transferase, and NAD(P)H dehydrogenase quinone 1 by binding the antioxidant response elements in the promoter regions of these genes (Mcmahon et al., 2003; Holguin, 2013).

**Figure 4:**
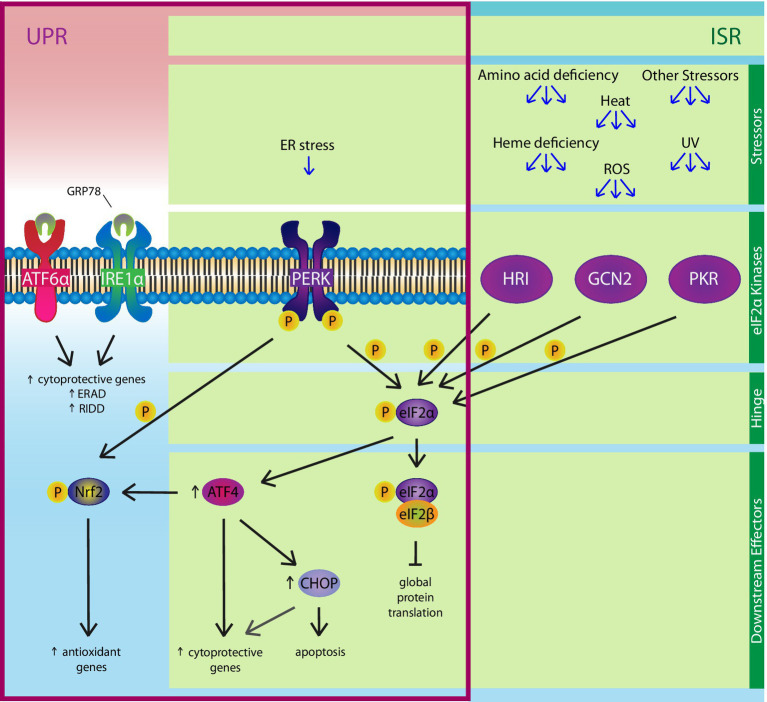
The Integrated Stress Response (ISR). The PERK pathway of the UPR is also a member of the ISR. Various stressors, including ER stress, amino acid deficiency, ultraviolet rays, heat, ROSs, and heme deficiency, can activate one or more of the four eIF2α kinases: PERK, HRI, GCN2, and PKR. The ISR hinges on eIF2α, which is phosphorylated by the four kinases. Phosphorylated eIF2α binds eIF2β, a key component of an essential complex involved in initiating protein translation, to inhibit global protein synthesis, except ATF4 and ATF4-regulated genes like CHOP. ATF4 positively regulates expression of cytoprotective genes, as well as upregulating CHOP, which can induce apoptosis under chronic ER stress conditions. Independent of the ISR, ER stress-induced activation of the PERK pathway can also enhance the anti-oxidant response by upregulating genes *via* the direct phosphorylation of nuclear factor erythroid 2-related factor (Nrf)2.

ER and oxidative stress are interconnected in their responses to physiological and pathological stressors. In fact, part of the anti-oxidant response and the UPR constitute pathways that comprise the integrated stress response (ISR), which hinges on the phosphorylation of eIF2α (Figure 4; Van’t Wout et al. 2014; Taniuchi et al., 2016). In eukaryotic cells, eIF2α is phosphorylated by four kinases, which are PERK, heme-regulated inhibitor (HRI) kinase, protein kinase R (PKR), and general control non-derepressible (GCN)2. While multiple stressors can activate the same kinase, a single stressor can also activate multiple kinases. In an *in vitro* study that knocked down expression of the four eIF2α kinases and re-introduced each kinase separately, the oxidative stress-inducers, H_2_O_2_ and carbonyl cyanide p-(trifluoromethoxy) phenylhydrazone, phosphorylated eIF2α through HRI kinase and GCN2 (Taniuchi et al., 2016). Once activated, P-eIF2α inhibits global protein synthesis, thereby decreasing demand for oxidative folding, which reduces ROSs formed as a byproduct of the reaction and attenuates oxidative stress. P-eIF2α also reduces oxidative stress by upregulating ATF4, a mediator that has been shown to enhance Nrf2 recruitment to the anti-oxidant response element of oxidative stress-responsive genes like heme oxygenase-1 (He et al., 2001; Harding et al., 2003; Mimura et al., 2019). Finally, there is an eIF2α-independent mechanism through which the UPR regulates the antioxidant response. During ER stress, Nrf2 is directly phosphorylated by PERK, allowing its release from Keap1 so it can translocate to the nucleus where it upregulates expression of anti-oxidant genes (Cullinan et al., 2003; Cullinan and Diehl, 2004). Altogether, the UPR acts as a positive regulator of the antioxidant response *via* P-eIF2α inhibition of protein translation, ATF4 enhanced recruitment of Nrf2 to antioxidant response elements, and PERK phosphorylation of Nrf2.

The redox potential of the cell and particularly that of the mitochondrion is tightly controlled and is essential for metabolism, cell growth and differentiation. When dysfunctional, oxidative stress may lead to mitophagy and autophagy. Cellular responses are initiated in the face of excessive oxidative stress in order to avoid these consequences and to promote cell survival (Dodson et al., 2013). Oxidative stress can interfere with the oxidative folding of unfolded proteins to induce ER stress. Correspondingly, dysregulated S–S formation in ER stress can allow ROSs to accumulate in the cell where they contribute to oxidative stress (Tavender and Bulleid, 2010). Most pathological airway studies on the topic focus on understanding the former and generally conclude that inhibiting oxidative stress (i.e., with antioxidants) leads to reduced ER stress, which decreases UPR activation and coincides with improved outcomes (Tagawa et al., 2008; Kenche et al., 2016; Wang et al., 2017a). However, consistent with topics covered in this review, we have focused on the latter, which is to examine the role of ER stress and the UPR on lung structure and function, in this case the antioxidant response in the lungs.

In a simple model of oxidative stress-induced airway injury, like hyperoxia, there is no concrete evidence of UPR activation that cannot also be attributed to the ISR, which shares the eIF2α-ATF4-CHOP axis (Figure 4). For example, in a murine model of hyperoxia-induced acute lung injury, CHOP expression increased, correlating with increased lung permeability and edema (Lozon et al., 2011). However, the expression of CHOP was confirmed to be downstream of the ISR eIF2α kinase, PKR, and not PERK. Interestingly, CHOP^−/−^ mice were more sensitive to hyperoxia-induced acute lung injury than wild type mice and had a higher rate of mortality, indicating that CHOP expression is protective in this model. This could be the result of CHOP regulation of genes besides those related to apoptosis, which could be attributed to differences in the mechanism of CHOP activation, in this case by PKR (or HRI and GCN2) vs. PERK (Vij et al., 2008; Lozon et al., 2011; Yang et al., 2017). In other studies, hyperoxia attenuated the expression of UPR mediators GRP78 and PDIA3 (Gewandter et al., 2009; Xu et al., 2009). Both the overexpression and inhibition of GRP78 had no effect on ROS production or UPR activation, while overexpression and siRNA knockdown of PDIA3 increased and decreased hyperoxia-induced apoptosis of endothelial cells, respectively. Altogether, these studies indicate that ER stress and the UPR do not play significant roles in hyperoxia-induced airway injury, while activating the UPR in a model of disease without ER stress could aggravate rather than ameliorate oxidative stress-induced airway injury.

Expanding on our understanding of ER stress and the UPR in disease, we investigated their roles in complex models of oxidative stress-induced airway injury in which ER stress was also induced. *In vivo* and *in vitro* exposure to cigarette smoke extract is known to induce both stress responses (Lin et al., 2017b, 2019). Raising the protein folding capacity of lung cells by administering an ER stress inhibitor/chemical chaperone reduced cigarette smoke extract-induced airway remodeling and emphysema in the rat, which coincided with an augmentation in the antioxidant response (Lin et al., 2019). In a bleomycin-induced model of fibrosis, the adoptive transfer of mesenchymal stem cells reduced airway fibrosis and attenuated ER stress through PERK-Nrf2, but not the PERK-eIF2α-ATF4-CHOP pathway, suggesting that the ER stress-induced activation of the non-canonical PERK-Nrf2 pathway of the UPR may have a protective role in complex airway diseases (Ono et al., 2015; Lee et al., 2020). Similarly, activation of the PERK-Nrf2 pathway was suppressed in immortalized AECs, as well as blood cells and lung tissues from patients with CF and reversal of the pathway by salubrinal decreased inflammatory responses to flagellin and *P. aeruginosa* (Blohmke et al., 2012). Finally, the neutrophilic inflammation and edema that characterized lipopolysaccharide-induced acute lung injury were ameliorated through the PERK-Nrf2 pathway using the plant-derived alkaloid berberine (Liang et al., 2019). Thus, in contrast to hyperoxia-induced airway injury, disease outcomes could be improved by inhibiting ER stress or activating the PERK-Nrf2 pathway in complex airway diseases. Unfortunately, there are few other studies addressing the role of ER stress in airway diseases where the antioxidant response was is measured.

### Bronchomotor Tone

Airway smooth muscles (ASMs) constrict in response to contractile agonists, which are the primary elements that increase bronchomotor tone and subsequently limit airflow (Martin et al., 2000). Pathological alterations in ASM characteristics have been extensively documented in airway inflammatory diseases, especially asthma and COPD (Bosken et al., 1990; Ozier et al., 2011). The increases in ASM mass observed in both diseases are likely the combined result of ASM cell (ASMC) hypertrophy and hyperplasia (Bosken et al., 1990; Ozier et al., 2011). These changes are proposed to contribute to overall elevated force generation and worsened airway narrowing (Lambert et al., 1993). The biological mechanisms mediating ASM remodeling are not fully elucidated and the precise role of ER stress is unknown.

It has been established that the phenotypes of smooth muscle cells in general display a dichotomy of either contractile or proliferative/secretory characteristics (Dekkers et al., 2012). Current evidence suggests that growth factors and inflammatory mediators in diseased airways promote the conversion of ASM to the proliferative phenotype and induce hyperplasia (Bentley and Hershenson, 2008). Pathways related to ER stress could dependently or independently participate in such processes, but there is as yet no direct evidence showing the relationship between ER stress and ASMC properties. However, research on other smooth muscles suggests that ER stress in general can act as a promoter of the proliferative smooth muscle phenotype. For example, fibroblast growth factor-2 upregulates ATF4 expression, which is directly responsible for inducing rat vascular smooth muscle proliferation (Malabanan et al., 2008). Platelet-derived growth factor also activates the IRE1α-XBP1 pathway of the UPR in vascular smooth muscle cells and drives proliferation *via* the downregulation of calponin h1 (Zeng et al., 2015). Vascular smooth muscle cells from mice with smooth muscle-specific knock-out of CHOP exhibit decreased proliferation induced by atherosclerotic lesions, due to the accumulation of the growth-inhibiting factor KLF4 (Zhou et al., 2015).

A recent study featuring the analysis of existing gene array data revealed an inverse relationship between the *in vivo* expression of ER stress markers and contractile proteins in human bladder smooth muscle and arterial smooth muscle (Zhu et al., 2020). The induction of ER stress in human bladder smooth muscle cells, using dithiothreitol or Tm, reduced contractile protein expression in an IRE1α-XBP1-dependent manner. Conversely, overexpression of myocardin, a master transcription factor of contractile genes and myocardin-related transcription factor suppressed the induction of ER stress. Though the proliferative properties of smooth muscle were not investigated, this study hinted that ER stress demonstrates a mutually antagonistic relationship with the contractile phenotype, potentially inducing pathological changes in smooth muscle. There is a potential that increased ER stress participates in the induction of the proliferative phenotype in ASMs through these processes, leading to remodeling and associated pathophysiology. Experimental evidence specifically conducted on ASM is required to confirm this hypothesis.

ASMs respond to a large variety of inflammatory mediators, which could induce ER stress. Notably, TNFα selectively activates the IRE1α-XBP1 pathway in cultured ASMCs but not the PERK or ATF6 pathways, which can be inhibited by the superoxide scavenger, tempol (Yap et al., 2020). IRE1α activation subsequently results in Mfn2 downregulation, a factor responsible for mitochondrial fusion and tethering to the ER, leading to an increase in mitochondrial fission (Delmotte and Sieck, 2019). Since mitochondria tethered to the ER absorb Ca^2+^ during its release from the ER and act as a buffering agent to control cytosolic [Ca^2+^], the authors also argue that the loss of Mfn2 due to IRE1α-XBP1 activation is responsible for TNFα-mediated mitochondrial dissociation from the ER (Delmotte et al., 2017). This corresponds to impaired Ca^2+^-buffering in the mitochondria and leads to increased cytosolic Ca^2+^ influx upon contractile agonist stimulation, subsequently contributing to the increased contractility of ASMCs (Delmotte and Sieck, 2019).

Tm-induced ER stress in murine ASMCs initiates synthesis of hyaluronan, an ECM protein observed in higher abundance within the asthmatic airway submucosa (Roche et al., 1989; Lauer et al., 2009). In this manner, ASMs secrete ECM proteins that directly contribute to the remodeling of the extracellular environment (Bourke et al., 2011). Conversely, ASMCs sense the ECM environment, which alters their contractile/proliferative phenotypes (Freyer et al., 2001). This may be accomplished through the increased infiltrative capacity of leukocytes as high hyaluronan content in the ECM has been shown to enhance leukocyte adhesion to ASM-derived matrix (Lauer et al., 2009). Mast cells and CD4^+^ T cells have been observed to infiltrate ASM bundles in higher numbers in asthmatics, where they potentially mediate some of the functional changes associated with asthma pathophysiology (Brightling et al., 2002; Ramos-Barbon et al., 2010). T cells can exert pro-proliferative effects on ASMCs *via* hyaluronan-specific binding (Al Heialy et al., 2013). Altogether, these data indicate that ER stress in ASMs play a role in ECM remodeling and the ECM can in turn enhance recruitment of leukocytes to ASMs where they induce ASMC proliferation.

### Airway Inflammatory Response

The inflammatory response is a physiological response to injury. Inflammatory cells, including macrophages, eosinophils, neutrophils, and lymphocytes, are cells that migrate to the site of injury where they interact directly with the source of injury or infection and release mediators that coordinate the removal of harmful stimuli and initiate repair (Aghasafari et al., 2019). However, on occasion, the response does more damage than good, as is the case with some airway inflammatory diseases, such as COPD and asthma. The inflammatory profile of a disease can also vary based on the type of insult or injury, its duration, as well as genetic and epigenetic factors, health history, and condition of the host (Perez-Novo and Bachert, 2015; Wesolowska-Andersen and Seibold, 2015).

The immune response to injury almost always induces some degree of ER stress since among other considerations, inflammatory cytokines and chemokines rely heavily on the ER for their maturation; proliferating (immune) cells double their protein content before undergoing cell division; and *de novo* protein synthesis is essential for tissue repair and cell differentiation in response to injury (Iwakoshi et al., 2003b; Brunsing et al., 2008; Waldschmitt et al., 2014). Nevertheless, while ER stress is induced in airway inflammatory disease, less is known of the specific roles of the three canonical pathways of the UPR. Here, we address the role of the UPR in immune cell development, maturation, differentiation, and function. We also explore the profiles of UPR activation in the context of airway inflammatory disease and injury.

The highly conserved, IRE1α-XBP1 axis is the best studied of the three pathways of the UPR and is the most critical to the development, maturation, differentiation, survival, and function of most hematopoietic cells. A study looking at temporal changes in activity determined that the IRE1α-XBP1 pathway is active at early stages of T-lymphocyte development and differentiation, including CD4^+^CD8^+^ (double positive) thymic T cells, compared to mature T cells (Brunsing et al., 2008). IRE1α-XBP1 is also activated in CD8^+^ T cells, in response to bacterial and viral infections and the pathway plays an important role in terminal effector functions (Kamimura and Bevan, 2008). In CD4^+^ Th2 cells, the inhibition of IRE1α attenuates the secretion of interleukin (IL)-5, but not IL-4 (Poe et al., 2019). IL-5 is still produced, but is retained within the cell, indicating that IRE1α is specifically involved in the PTM and maturation of IL-5 that is required for its release. This pathway is also active at early stages of B-lymphocyte differentiation, including pro-B cells in the bone marrow and is less active in mature B cells (Brunsing et al., 2008). It is not essential for B cell cytokine production or survival, but is required for the terminal differentiation of plasma cells and the production and secretion of immunoglobulin M (Reimold et al., 2001; Iwakoshi et al., 2003a,b; Tirosh et al., 2005). The IRE1α-XBP1 pathway may be essential for early stage dendritic cell (DC) development, survival, and type-I interferon production in response to TLR9 agonists, but appear to be less important in committed CD11c-expressing DCs (Iwakoshi et al., 2007; Osorio et al., 2014). In granulocytes, XBP1 is required for eosinophil development, differentiation, and survival, in addition to the production of eosinophil granules (Bettigole et al., 2015). Although XBP1 is dispensable for neutrophil and basophil survival, an *in vitro* study using a human leukemia cell line shows that IRE1α activity is increased in differentiating neutrophils, while ATF6α and PERK activity are suppressed (Bettigole et al., 2015; Tanimura et al., 2018). Finally, an inhibitor of IRE1α kinase activity was shown to induce cell death in a mast cell leukemia cell line, indicating that this pathway may be important in mast cell survival (Mahameed et al., 2019). Altogether, IRE1α and its downstream mediators appear to be critical to the proper development, survival, and function of most, if not all, hematopoietic cells.

Apart from the IRE1α pathway, there is a significant gap in our understanding of the role of the UPR in inflammatory cell development and function. What is known is that differentiating macrophages have been shown to upregulate expression of the ER chaperones, GRP78 and GRP94, in addition to XBP1s (Dickhout et al., 2011). Macrophages may also rely on ER stress to differentiate into the M2 phenotype as the ER stress inhibitor, phenylbutyric acid, was shown to inhibit M2 differentiation (Oh et al., 2012). Although the precise arms of the UPR involved in regulating the M2 phenotype is unclear, there is evidence of both IRE1α and PERK activity. Similarly, the IRE1α and PERK pathways have been implicated in mast cell survival and DC production of IL-23 (Goodall et al., 2010; Marquez et al., 2017; Mahameed et al., 2019). GRP94-deficient B cells can survive, develop and even function properly (Randow and Seed, 2001). However, these cells produce significantly fewer antibodies following TLR activation and have defects in integrin formation (Melnick et al., 1992; Randow and Seed, 2001; Liu and Li, 2008; Wu et al., 2012; Pagetta et al., 2014). GRP78 is crucial for the assembly of immunoglobulin chains, binding the H and L domains, and it binds the TCR until assembly partners can come in to complete assembly (Haas and Wabl, 1983; Hendershot, 1990; Melnick et al., 1992; Vanhove et al., 2001). In hematopoietic stem cell progenitors, experiments in which the ER chaperone, CRT, was overexpressed or silenced indicated that CRT may be essential in the differentiation of erythroid cells and megakaryocytes (Salati et al., 2017). These studies indicate that the UPR and its mediators are important and even central to the maturation and function of many immune cells, which could make them ideal candidates for targeted therapy in complex diseases.

In previous sections, we addressed AECs and their importance in maintaining a physical barrier between the environment and the inner milieu and in MCC. However, AECs are also important participants in innate immune responses. These cells represent the first line of defense against harmful pathogens. Several chronic airway inflammatory diseases have been associated with increased epithelial proinflammatory cytokine production (Machen, 2006). There may also be evidence of ER stress; for example, airway infections activate XBP1 and increase Ca^2+^ stores to amplify Ca^2+^-dependent IL-8 secretion *in vitro* (Martino et al., 2009). Human epithelium in CF patients show higher IRE1α/XBP1 activation by ER stress and induces cytokine production (Hull-Ryde et al., 2021). ER stress boosts TLR-mediated IL-6 and IL-8 expression and secretion *via* PERK-and ATF6-mediated p38 and ERK activation in human primary bronchial epithelial cells (Mijosek et al., 2016). Additionally, house dust mite-induced ATF6 activation is associated with AEC death, hyperresponsiveness and subsequent airway fibrosis in mice (Hoffman et al., 2013). It also increases the production of IL-25, which increases CHOP and P-PERK expression and induces epithelial tight junction injury and cell apoptosis in human bronchial epithelial cells (Yuan et al., 2018). Cigarette-smoke increases the expression of CHOP, caspase-12 (an ER stress-induced mediator of apoptosis), and other markers of apoptosis in rat lungs. The nicotine component of cigarette smoke also increases the expression of CHOP, caspase-12, and apoptosis in human bronchial epithelial cells (Lin et al., 2017a). In infection, influenza A virus (IAV)-induced ER stress activates ATF6, but not CHOP. This activation of the ER stress response induces caspase12–dependent apoptosis of and TGFβ production by murine epithelial cells (Roberson et al., 2012). Deletion of *Grp78* in alveolar type 2 cells in mice results in ER stress, apoptosis, senescence, and activation of TGFβ, with resulting lung fibrosis (Borok et al., 2020).

In inflammatory diseases of the airways, mechanisms that reduce ER stress and/or enhance UPR activation generally improve outcomes, including asthma. Asthma is a heterogeneous and complex disease in which the UPR is activated in response to the ER stress in the lungs (Pathinayake et al., 2018). Further enhancement of ER stress in an allergen-induced model of asthma by Tm administration increases airway cytokine production, inflammation, and AHR (Guo et al., 2017). In contrast, the attenuation of ER stress in murine models of asthma, *via* the administration of ER stress inhibitors like tauroursodeoxycholic acid, the epithelium-specific ablation of PDIA3, or the siRNA-targeted inhibition of PDIA3 and ATF6, attenuate allergen-induced ER stress, AHR, inflammation, and fibrosis (Hoffman et al., 2016; Siddesha et al., 2016; Nakada et al., 2019). In a genome-wide association study, the ORMDL3 (ORMDL sphingolipid biosynthesis regulator 3) gene was identified as having a strong association with asthma (Moffatt et al., 2007). This gene regulates ER stress by regulating Ca^2+^ signaling and increased expression leads to an attenuation of ER-mediated Ca^2+^ signaling and increases activation of the UPR, specifically activating the ATF6α arm (Cantero-Recasens et al., 2010; Miller et al., 2014). ORMDL3-deficient mice are protected in a murine model of asthma with reduced AHR, lung eosinophils, allergen-specific serum IgE, and IL-6 in response to the fungus, *Alternaria alternata*, while overexpression of ORMDL3 enhanced AHR in this model (Loser et al., 2017). Additionally, ORMDL3, which is predominantly expressed in AECs, is strongly associated with AHR, as well as airway remodeling, inflammation, and mucus hypersecretion, in other allergen-models of asthma (Miller et al., 2012, 2014; Oyeniran et al., 2015).

Several UPR-related mediators are upregulated in the lungs of tobacco smokers compared to non-smokers, including GRP78, CRT, and PDIA1 (Kelsen et al., 2008). Cigarettes are a major risk factor for the development of COPD, of which emphysema and chronic bronchitis are the most common features. An abnormal inflammatory response and ER stress are both characteristic of the disease with reports of increased expression of ERAD proteins, ubiquitinylated proteins, and accumulation of misfolded protein aggregates in the lungs of patients with severe emphysema (Min et al., 2011). Cigarette smoke affects the protein-folding capacity of the cell by damaging proteins involved in the proper folding of nascent proteins. For example, the lungs of mice exposed to cigarette smoke have 4–6 times higher oxidized and sulfenylated PDIA1 compared to control mice, but are completely deficient in the reduced form of PDIA1 (Kenche et al., 2013, 2016). A deficiency in the reduced form of PDIs adversely affects the isomerization of S–Ss as they require their reductase activity to break the initial bonds before rearrangement. Moreover, most PTMs to PDIs, including highly oxidized or sulfenylated PDIs, are associated with reduced enzymatic activity. Cysteine and tyrosine modifications to PDIA1 by cigarette smoke extract and some of its known pro-oxidative components like H_2_O_2_, peroxynitrite (a reactive nitrogen species), acrolein and hydroxyquinone (long lived free radicals) can alter PDIA1 structure and function, which effectively reduces overall protein folding, activates the UPR, and decreases chaperone, reductase, and isomerase activity (Kenche et al., 2016).

Strategies that reduce ER stress in the lungs of animals exposed to cigarettes have reduced airway inflammation, apoptosis, and remodeling (Lin et al., 2017b; Wang et al., 2017b; Lin et al., 2019). IRE1α, CHOP, and GRP78 that are upregulated in the lungs of cigarette smoke-exposed mice are reduced in mice treated with the ER stress inhibitor, 4-phenylbutyric acid (Wang et al., 2017b). Moreover, BALF chemokines and inflammatory cell numbers, including neutrophils, are significantly reduced in these mice. In another study, the overexpression of GRP78 and inhibition of the downstream, apoptosis-inducing UPR transcription factor, CHOP, significantly reduced cigarette smoke extract-induced apoptosis in an AEC line, further indications that enhancing the protein folding capacity of cells may be protective against cigarette smoke-induced airway injury and progression of COPD (Tagawa et al., 2008).

Patients with cystic fibrosis are prone to chronic airway infection and a corresponding and sustained inflammatory response that is detrimental to lung structure, health and function. The CF lung has significantly greater inflammation than the normal lung, which is associated with the activation of the IRE1α arm of the UPR (Lara-Reyna et al., 2019). IRE1α is upregulated in innate immune cells, mainly monocytes, neutrophils, and M1 macrophages. Activation of IRE1α leads to an increase in the metabolic activity of M1 macrophages from CF patients and its inhibition significantly reduces production of the inflammatory cytokines, IL-6 and TNFα. There is also an absence of PERK-eIF2α activity in the CF lung and in cultured AECs from ΔF508-CFTR patients, but activation of this pathway using an eIF2α agonist beneficially attenuated the robust inflammatory response to flagellin and *P. aeruginosa* (Nanua et al., 2006; Blohmke et al., 2012).

### Viral Infection

The ER is central to the processing of proteins required to mount an effective host immune response to infection, but in many cases viruses like HIV, IAV, and hepatitis C virus hijack the host ER machinery to translate, fold and package structural proteins for virion production (Bagchi, 2020). The IAV structural glycoprotein, hemagglutinin (HA), coordinates viral fusion and entry into target cells, preferentially binding the α2,6- and α2,3- linked sialic acid receptors, which are expressed in abundance on the human airway epithelium (Couceiro et al., 1993; Shinya et al., 2006). During replication, HA interacts with ER chaperones like CNX and CRT for proper folding (Hebert et al., 1997). Additionally, PDIs are essential for the efficient oxidative folding of viral proteins, including PDIA3 on HA of IAV, PDIA1 on the E1 and E2 glycoproteins of hepatitis C virus, and PDIA3 on the F proteins of respiratory syncytial and Sendai viruses (Solda et al., 2006; Kim and Chang, 2018; Ozcelik et al., 2018; Piacentini et al., 2018). PDIA3 forms S–Ss between cysteine residues in HA and is significantly upregulated in mouse lungs following infection by various strains of IAV, as well as in human lung epithelial cells following infection with a pandemic, although not a seasonal strain of influenza (Chamberlain et al., 2019). Furthermore, epithelial-specific PDIA3 knockout mice have significantly lower viral burdens, less inflammation and better lung function. In addition, the ER stress inhibitors, tauroursodeoxycholic acid and 4-phenylbutyrate, are effective at, respectively, reducing the expression of viral proteins in human tracheobronchial epithelial cells and lowering the viral titer in the airways of mice infected with IAV (Hassan et al., 2012; Jung et al., 2019).

Infection by the respiratory virus, coronavirus (CoV) infectious bronchitis virus, activates the PERK pathway, but siRNA knockdown of the downstream mediator, CHOP, reduced apoptosis of infected cells and inhibited viral replication (Liao et al., 2013). Interestingly, primary bronchial epithelial cells from CF patients show less evidence of ER stress following rhinovirus infection than cells from healthy donors and therefore activate the UPR to a lesser degree (Schogler et al., 2019). In CF cells, the induction of ER stress with Tm or other chemical stressors is extremely effective at reducing rhinovirus replication and shedding. Altogether, these studies show a wide range of ER stress responses and patterns of UPR activation, but also highlight the therapeutic potential of targeting the UPR in viral infection.

In relation to ERAD, this IRE1α-XBP1-mediated pathway counters viral infections by degrading unfolded viral proteins, thereby limiting viral replication. This pathway has been shown to be activated in mouse embryonic fibroblasts and in a human alveolar epithelial cell line, in response to IAV infection (Frabutt et al., 2018; Jung et al., 2019). The HA glycoprotein alone is highly effective at activating IRE1α and the ERAD machinery (Frabutt et al., 2018). However, some viruses manipulate ERAD and secretory pathways to evade the host immune response by suppressing the expression of viral proteins on the cell surface where they can be recognized by immune cells, such as natural killer cells (Wilkinson et al., 2008). The human cytomegalovirus targets the major histocompatibility class I polypeptide-related sequence A (MICA), a stress-induced protein that is upregulated on the cell surface of virus-infected cells (Seidel et al., 2021). Specific receptors on natural killer cells then recognize this stress-induced ligand, allowing it to be targeted for elimination. During human cytomegalovirus infection, the signal peptide on the viral glycoprotein, US9, which has an unusually slow rate of cleavage, sustains its presence in the ER where it targets MICA for proteosomal degradation before it can be expressed on the surface of the cell.

Although GRP78 is largely localized to the ER, under ER stress conditions, a small fraction of the chaperone is translocated to the cell surface (Elfiky et al., 2020). Cell surface-GRP78 is upregulated in many cancer cells, including breast and prostate cancers and has become a target for cancer therapy (Tsai and Amy, 2018), In infection, cell surface-GRP78 can assist viral attachment and entry into the cell by binding pathogenic proteins, including the spike (S) protein on the outer envelope of viruses and coat proteins on fungi (Elfiky et al., 2020). Cell surface-GRP78 is expressed on several mammalian cells, including the human airway cell lines, A549, Beas2B, and Calu3 and is upregulated by a variety of viruses (Nain et al., 2017; Chu et al., 2018; Elfiky et al., 2020) The receptor-binding domain of the S protein of different members of the CoV family can interact with angiotensin-converting enzyme-2 (ACE2), dipeptidyl peptidase-4, and cell surface-GRP78, allowing the membranes of the virus and target cell to fuse (Chu et al., 2018; Allam et al., 2020). In Middle East Respiratory Syndrome (MERS)-CoV, cell suface-GRP78 does not independently allow nonpermissive cells to be infected by the virus, but facilitates entry of the virus into permissive cells in the presence of dipeptidyl peptidase-4 (Chu et al., 2018). In line with other CoVs, modeling studies predict cell surface-GRP78 binding to the receptor-binding domain of the S protein of Severe Acute Respiratory Syndrome (SARS)-CoV-2, the virus causing COVID-19 (Ibrahim et al., 2020). Moreover, the GRP78 binding site is predicted to overlap with the binding site of the ACE2 receptor, evidence that GRP78 may be a receptor directly utilized by SARS-CoV-2 to infect target cells (Aguiar et al., 2020). Serum GRP78 levels are also reported to be higher in COVID-19 positive patients compared to COVID-19 negative patients with pneumonia and healthy controls (Sabirli et al., 2021). Several candidate peptides and small molecules targeting the GRP78-binding site on the S protein of SARS-CoV-2 and the viral docking site on GRP78 have been identified, of which Satpdb18674 and epigallocatechin gallate are predicted to be the most effective (Allam et al., 2020). As of yet, no follow up studies have been performed to experimentally confirm the effectiveness of targeting the GRP78-S protein binding sites to inhibit SARS-CoV-2 infection and reduce viral load.

The spike protein of SARS-CoV-2 is synthesized in the ER of the infected cell where it undergoes protein modifications, including a predicted 22 N-and O-linked glycosylation sites on the S protein, before undergoing trimerization and further processing in the Golgi (Zhang et al., 2021). The receptor-binding motif and receptor-binding domain of the S protein of SARS-CoV-2 contain 1 and 3 S–Ss, respectively (Lan et al., 2020). They interact with ACE2 for cell entry and reducing S–Ss into thiols on the S protein and/or ACE2 are predicted to significantly impair binding and therefore infectivity (Hati and Bhattacharyya, 2020). Once again, the implications are that inhibitors of PDIs, PTMs, and ER stress have therapeutic potential as inhibitors of viral infection, including the COVID-19 causing SARS-CoV-2.

## Summary

Upon the induction of ER stress, GRP78 dissociates from and activates the three key receptors that orchestrate the UPR, ATF6α, IRE1α and PERK. ATF6α is proteolytically cleaved to liberate its transcriptional factor domain, which in turn upregulate genes associated with the protein folding machinery. The endonuclease activity of IRE1α allows for its dual function of initiating RIDD, as well as activating XBP1 by alternative splicing of its mRNA, which promotes the expression of similar protein folding-associated genes. The receptor, along with unfolded proteins are degraded *via* ERAD, in an effort to reduce the source of ER stress. PERK phosphorylates eIF2α, which orchestrates the reduction in protein synthesis, promoting cytoprotective responses *via* ATF4-mediated transcriptional regulation and inducing apoptosis through the activation of CHOP in response to chronic ER stress. In addition, the ability of PERK to directly activate eIF2α and Nrf2 makes it a part of the ISR, which corresponds to signaling beyond the UPR, such as the activation of antioxidant responses, to restore cellular homeostasis. The PTM of peptides is also an important task of the ER, and dysregulation in the machinery involved in signal peptide cleavage, glycosylation and S–S formation will activate the UPR.

ER stress is associated with the pathogenesis of various lung diseases, and is associated with structural cell damage, dysfunction, and the inflammatory response. However, ER stress is not always pathological in that it plays an important role in immune cell development, cell division and other functions, when accompanied by a sufficient and appropriate UPR. Although it is not always clear whether ER stress is a cause or consequence of pathology, inhibiting ER stress and/or activating the UPR, in certain contexts, have some demonstrated therapeutic benefits. This is likely attributed to overlaps in stress responses and their pathways, including the pathways of and genes regulated by the three canonical UPR receptors, the ISR pathways hinging on eIF2α, and the many mediators that make up the protein-folding machinery in the ER like the chaperone and S–S-forming functions shared by many PDIs. Equally as important as the therapeutic potential of targeting the UPR is in its potential to induce cellular apoptosis. Thus, ER stress inhibitors, UPR and ISR activators, and other chemical modifiers affecting protein folding and degradation could in some cases augment rather than attenuate disease. It would be prudent to evaluate this approach as a therapy, on a case-to-case basis. Continued investigation into the roles of ER stress, the UPR and protein processing as they apply to the pathophysiology of pulmonary disease will provide us with a deeper understanding of how to navigate these complex diseases.

## Author Contributions

EN, RS, and UF wrote the first draft of the manuscript. EN and JM contributed to the initial conception and layout of the review and edited the manuscript. All authors contributed to the article and approved the submitted version.

### Conflict of Interest

The authors declare that the research was conducted in the absence of any commercial or financial relationships that could be construed as a potential conflict of interest.

## Abbreviations

AEC: Airway epithelial cell
AGR: Anterior gradient
AHR: Airway hyperresponsiveness
ASM: Airway smooth muscle
ASMC: Airway smooth muscle cell
ATF4: Activating transcription factor 4
ATF6α: Activating transcription factor 6α
BCL2: B cell lymphoma 2
CF: Cystic fibrosis
CFTR: Cystic fibrosis transmembrane conductance regulator
CHOP: C/EBP Homologous Protein
CNX: Calnexin
CoV: Coronavirus
CRT: Calreticulin
DC: Dendritic cell
ECM: Extracellular matrix
eIF2α: Eukaryotic initiation factor-2α
ER: Endoplasmic reticulum
ERAD: ER-associated degradation machinery
ERO1α: ER oxidoreductase 1α
GCN2: General control nonderepressible 2
GRP78: Glucose-regulated protein 78 kDa
HA: Hemagglutinin
HIV: Human immunodeficiency virus
HRI: Heme-regulated inhibitor
IAV: Influenza A virus
IPF: Idiopathic pulmonary fibrosis
IRE1α: Inositol-requiring enzyme 1α
Keap1: Kelch-like ECH associated protein 1
MCC: Mucociliary clearance
MICA: Major histocompatibility class I polypeptide-related sequence A
Nrf2: Nuclear factor erythroid 2-related factor 2
ORF: Open reading frame
P: Phosphorylated
PDI: Protein disulfide isomerase
PDIA: Protein disulfide isomerase family A
PERK: Protein kinase R-like ER kinase
PKR: Protein Kinase R
PTM: Post translational modification
RIDD: Regulated IRE1-dependent decay
ROS: Reactive oxygen species
S: Spike
S1P: Site-1 protease
S2P: Site-2 protease
–SH: Thiol/sulfhydryl side chain
SP: Signal peptide
S–S: Disulfide bond
Tm: Tunicamycin
Trx: Thioredoxin
UPR: Unfolded protein response
XBP1: X-box binding protein-1
